# Identification of interferon-stimulated genes with modulated expression during hepatitis E virus infection in pig liver tissues and human HepaRG cells

**DOI:** 10.3389/fimmu.2023.1291186

**Published:** 2023-11-20

**Authors:** Léa Meyer, Isoline Duquénois, Stacy Gellenoncourt, Marie Pellerin, Aïlona Marcadet-Hauss, Nicole Pavio, Virginie Doceul

**Affiliations:** Institut National de Recherche pour l’Agriculture, l’Alimentation et l’Environnement (INRAE), Agence Nationale de Sécurité Sanitaire de l’Alimentation, de l’Environnement et du Travail (ANSES), École Nationale Vétérinaire d'Alfort (ENVA), UMR Virology, Maisons-Alfort, France

**Keywords:** hepatitis E virus (HEV), interferon (IFN), IFN-stimulated gene (ISG), antiviral response, zoonosis

## Abstract

**Introduction:**

Hepatitis E virus (HEV) is a common cause of enterically transmitted acute hepatitis worldwide. The virus is transmitted by the fecal-oral route via the consumption of contaminated water supplies and is also a zoonotic foodborne pathogen. Swine are the main reservoir of zoonotic HEV. In humans, HEV infection is usually asymptomatic or causes acute hepatitis that is self-limited. However, fulminant hepatic failure and chronic cases of HEV infection can occur in some patients. In contrast, HEV infection in pigs remains asymptomatic, although the virus replicates efficiently, suggesting that swine are able to control the virus pathogenesis. Upon viral infection, IFN is secreted and activates cellular pathways leading to the expression of many IFN-stimulated genes (ISGs). ISGs can restrict the replication of specific viruses and establish an antiviral state within infected and neighboring cells.

**Methods:**

In this study, we used PCR arrays to determine the expression level of up to 168 ISGs and other IFN-related genes in the liver tissues of pigs infected with zoonotic HEV-3c and HEV-3f and in human bipotent liver HepaRG cells persistently infected with HEV-3f.

**Results and discussion:**

The expression of 12 and 25 ISGs was found to be up-regulated in infected swine livers and HepaRG cells, respectively. The expression of CXCL10, IFIT2, MX2, OASL and OAS2 was up-regulated in both species. Increased expression of IFI16 mRNA was also found in swine liver tissues. This study contributes to the identification of potential ISGs that could play a role in the control or persistence of HEV infection.

## 1 Introduction

Hepatitis E virus (HEV) is responsible for hepatitis E in human and belongs to the Paslahepevirus genus within the Hepeviridae family (1). Its genome is composed of a single stranded positive RNA that is 7.2 kb in length and codes for 3 open reading frames (ORF1 to 3) (2). *ORF1* codes for a non-structural polyprotein composed of several functional domains including a methyltransferase, a helicase and a RNA-dependent RNA polymerase (RdRp) (3). *ORF2* codes for the capsid protein and *ORF3* for a multifunctional phosphoprotein. Liver is the main site of HEV replication. A quasi-enveloped form of HEV particles is released from hepatocytes and is found in the serum whereas naked virus particles are found in the bile and feces (4). Four main genotypes of HEV can infect humans (HEV-1 to HEV-4). Genotypes 1 and 2 (HEV-1 and HEV-2) infect exclusively humans in endemic regions and are transmitted via the fecal-oral route, through the consumption of contaminated water or soiled food. In contrast, genotypes 3 and 4 (HEV-3 and HEV-4) are detected in humans and other animal species worldwide and can be transmitted via direct contact with infected animals or the consumption of infected meat (5, 6). Domestic and wild swine are the main reservoirs of zoonotic HEV and can replicate the virus efficiently even if infection in these hosts is asymptomatic. In domestic swine, experimental infections with HEV-3 cause subclinical acute infections with viral shedding lasting from 7 to 50 days (7). Chronic infections can also occur in the context of co-infection with viruses impairing the immune response such as porcine reproductive and respiratory syndrome virus (8) or when pigs are treated with immunosuppressive drugs (9). In most human cases, HEV infection causes acute hepatitis that is self-limited. However, fulminant hepatic failure can occur in patients with underlying chronic liver disease, in the elderly and in pregnant women (HEV-1). Chronic cases of HEV infection (viremia lasting for at least 3 to 6 months) that can rapidly lead to cirrhosis and/or liver transplantation have also been reported in immunocompromised patients such as solid-organ transplant recipients and involve mainly HEV-3 and HEV-4 (10). Rare cases of chronic hepatitis E in immunocompetent patients have also been reported (11). Extrahepatic manifestations of acute HEV or chronic HEV infection, including neurological syndromes have also been reported (12). No specific treatment against HEV infection has been approved yet but ribavirin has been successfully used to control hepatitis E replication in some patients. However, this antiviral drug causes side effects and clearance of the virus can fail (13). No vaccine has been commercialized outside China (14).

Interferon (IFN) is the host first line of defense against pathogens. Its secretion leads to the expression of hundreds of IFN-stimulated genes (ISGs) that can establish an antiviral state within infected and neighboring cells. ISG-encoded proteins can have an intrinsic antiviral activity, act directly on different signaling cascades involved in the IFN system to enhance its action and/or play a role in other cellular responses such as apoptosis and recruitment of immune cells or can also have proviral activity (15). Some ISGs can function in a pan-viral manner while others interfere specifically with a virus or a viral family (16). Recent investigations also suggest that a prolonged IFN response can be linked to viral persistence, but it is not yet clear whether it is a cause or a consequence (17). Several studies have shown that HEV (HEV-1 to HEV-4) triggers an IFN response in different *in vitro* and *in vivo* models as well as in patients suffering from chronic hepatitis E (18–20). IFN inhibits HEV replication *in vitro* (21–25) and pegylated IFNs have been used successfully to treat HEV-infected patients (26, 27). However, several studies suggest that IFN has a moderate and delayed antiviral effect on HEV infection *in vitro* and in patients in comparison to hepatitis C virus (HCV) (24, 25, 28). Several antagonists of the IFN pathways encoded by HEV ORF1, ORF2 and ORF3 have been identified suggesting that HEV has evolved counteracting strategies to modulate the antiviral response to establish an efficient infection (22, 29–33).

Knowing which ISGs are differentially expressed upon infection is important to identify potential host factors involved in the control or persistence of the disease. However, such knowledge is still partial in the context of acute and chronic HEV infections in humans and lacking for other natural hosts of HEV such as swine.

In this study, we aimed to determine which IFN-regulated genes are differentially expressed during acute infection with zoonotic HEV (HEV-3c and HEV-3f) in liver tissues from domestic pigs, the natural host of HEV. We also investigated expression of ISGs during chronic infection with HEV-3f in human hepatic cells. A model of persistent HEV-3f infection in HepaRG cells, previously developed in our group, that are able to differentiate into both biliary and hepatocyte-like cells and support HEV replication and release of infectious virions was used (34). These cells express a similar pattern of functional TLR/RLR than primary human hepatocytes and are a good surrogate model to study interactions between hepatotropic viruses and the hepatocyte innate system (35). We have shown that HEV-3f replication is slow in HepaRG cells, reaches a plateau around day 90 post-infection and is then maintained for several months without clearance of the virus (34). Expression of 84 to 168 genes involved in the IFN response was quantified using customized quantitative PCR (qPCR) arrays in these 2 models. Analysis of these data have shown that expression of 12 and 25 ISGs was up-regulated during HEV infection in swine liver and HepaRG, respectively. The expression level of five of these genes, *CXCL10*, *IFIT2*, *MX2*, *OASL* and *OAS2*, was up-regulated in both species. The expression of *IFI16* was also found to be up-regulated in swine liver tissues. This study contributes to the identification of putative host factors that could play a role in the control of HEV infection and need to be further investigated. In the future, this could contribute to the discovery of novel drug targets.

## 2 Materials and methods

### 2.1 Cell culture

Undifferentiated human HepaRG™ cells were purchased from BIOPREDIC International. Cells were grown in “proliferation medium” consisting of William’s E medium with GlutaMAX™ (ThermoFisher Scientific) supplemented with 10% heat-inactivated fetal calf serum (FCS), 5 μg/ml insulin (Sigma-Aldrich), 5x10^−5^ M hydrocortisone hemisuccinate (Sigma-Aldrich) and 100 IU/ml penicillin and 100 µg/ml streptomycin. Cells were maintained at 37°C in 95% air/5% CO_2_. Confluent HepaRG monolayers were passaged every 2 weeks and medium was renewed every 2-3 days. For differentiation, HepaRG were seeded into 6-or 24- well plate and cultured in proliferation medium for 2 weeks. Medium was then replaced for 2 extra weeks by “differentiation medium” consisting of William’s E medium with GlutaMAX™ (ThermoFisher Scientific) supplemented with HepaRG™ Differentiation Medium with antibiotics (ADD720C, BIOPREDIC International) or HepaRG proliferation medium supplemented with 1.2% DMSO (Sigma-Aldrich).

### 2.2 Virus inoculation of HepaRG cells

A HEV-3f strain originating from a French patient suffering from acute autochthonous hepatitis E was used and has been described previously (GenBank under accession number JN906974) (34, 36). Supernatant from the 6^th^ passage of the virus in HepaRG cells was used in this study to perform all the infections in HepaRG cells. HepaRG cells were differentiated in 6-well plates as described above. Two days before infection, differentiation medium was replaced by proliferation medium. Cells were infected overnight with an HEV inoculum diluted in proliferation medium to a final volume of 1ml at a multiplicity of infection (MOI) of 10 or 100 genome equivalent (GE)/cell. The viral suspension was then removed and cells were washed three times in PBS before adding 2 ml of proliferation medium. Every 2 to 3 days, one-half (1 ml) of the culture medium was replaced with fresh proliferation medium and infection maintained for up to 100 days.

### 2.3 IFN treatment of HepaRG cells

HepaRG were differentiated as described in the previous paragraph. Two days before treatment, differentiation medium was replaced by proliferation medium. Cells were then treated overnight with 200 IU/ml of IFN-β1a (PBL Interferon Source, Piscataway, NJ, USA) diluted in proliferation medium. IFN treatment was performed using HepaRG cells prepared in the same conditions and timing as for HEV inoculation.

### 2.4 Pig liver samples

Samples were collected in a previous study (37). This experimental protocol was validated by the ethics committee (ComEth number 12-043) of the National Veterinary School of Alfort, the French Agency for Food, Environmental and Occupational Health & Safety, and University Paris 12 and has obtained formal approval (notice number 09/10/12-9). HEV-3c (GenBank accession number JQ953664) and HEV-3f (GenBank accession number JQ953666) viral suspensions were generated using fecal samples from infected pigs. Eight-week-old specific pathogen free Large-White piglets were infected intravenously with 10^6^ copies of HEV RNA or mock-infected with PBS (37). At the peak of excretion (8 days post-infection), liver tissues were snap-frozen in liquid nitrogen. Samples from 2 control pigs, 3 pigs infected with HEV-3c and 3 pigs infected with HEV-3f were available and analyzed in this study.

### 2.5 Viral RNA extraction from supernatant

Viral RNAs were extracted from 200μl culture supernatants using the QIAamp Viral RNA Mini kit or the MagMAX core nucleic acid purification kit (Thermo Fisher Scientific, Courtaboeuf, France) and the KingFisher instrument according to the manufacturer instructions as described previously (38, 39).

### 2.6 Total RNA extraction

HepaRG cells were washed 3 times in cold PBS and harvested. Total RNA was extracted using the RNeasy minikit (Qiagen) including a digestion step on column with DNase I (Qiagen) according to the supplier’s protocol. For pig samples, around 25 mg of liver was added to RLT buffer (Qiagen) supplemented with 1% β-mercaptoethanol and lysed using a Fast Prep 24 System (MP Biomedicals, Illkirch, France) in Lysis Matrix D tubes (MPBiomedicals, Illkirch, France). Total RNA was then extracted using the RNeasy minikit (Qiagen) including a digestion step on column with DNase I (Qiagen) according to the supplier’s protocol.

### 2.7 RT^2^ Profiler PCR array

Five hundred ng of RNA extracted from HepaRG cells or swine liver samples were transcribed with the RT^2^ First Strand Kit (SA Biosciences, Qiagen, Courtaboeuf, France). The RT^2^ Profiler PCR array “Human Housekeeping genes (PAHS-000ZF-2, Qiagen)” and “Pig Housekeeping genes” (PASS-000ZF-2, Qiagen) were first used according to the manufacturer’s instructions to determine suitable housekeeping genes to normalize the data. *ACTB*, *GAPDH*, *HSP90AB1* and *GUSB* were selected for the human arrays and *ACTG*, *GAPDH*, *PGK1* and *RPL13A* for the porcine arrays. For HepaRG samples, 2 customized PCR arrays were used: the “Human Type I Interferon Response RT² Profiler PCR Array” (CAPH13839-PAHS-016Z, Qiagen) modified to include the selected human housekeeping genes and a RT² Profiler PCR Array (CLAH31374, Qiagen) customized to detect the expression of ISGs showed to be up-regulated in hepatic cells following IFN-I stimulation according to the interferome v2.01 database (40). For pig liver samples, a customized RT^2^ Profiler PCR array (CLAS34508, Qiagen) was designed to study the expression of 89 porcine genes involved in the IFN response. Data were analyzed using the RT^2^ Profiler PCR Arrays & Assays Data Analysis software (Qiagen) and normalized using the housekeeping genes selected above. Fold change was calculated by using the ΔΔCT method (41). An arbitrary cut-off of 2 was applied to determine significant differences.

### 2.8 HEV quantification by real-time quantitative PCR

HEV RNA quantification was adapted from the method described by Jothikumar et al. (42) as described previously (43). The QuantiTect Probe RT-PCR kit (Qiagen) was used according to the manufacturer’s instructions using 2 μl of RNA (template), 0.25 mM reverse primer (5’-AGGGGTTGGTTGGATGAA-3’), 0.1 mM forward primer (5’-GGTGGTTTCTGGGGTGAC-3’) and 5mM probe (FAMTGATTCTCAGCCCTTCGC-MGB). A LightCycler 480 apparatus (Roche Molecular Biochemicals) was used for sample analysis. Reverse transcription was carried out at 50°C for 20 min, followed by denaturation at 95°C for 15 min. DNA was amplified with 45 cycles at 95°C for 10 s and 58°C for 45 s. Standard HEV RNA was obtained after *in vitro* transcription of a plasmid pCDNA 3.1 ORF2-3 HEV and used to generate standard quantification curves as described previously (43).

### 2.9 Quantification of cellular gene expression by RT-qPCR

Total RNA was extracted as described above. A second digestion step was performed using a TURBO DNase (Ambion) and the RNA cleaned up on a column using the RNeasy minikit (Qiagen). RT was done using 500 ng of RNA with PrimeScript Reverse Transcriptase (Takara Bio Inc.) according to the manufacturer’s instruction. RT-qPCR was performed on 2 µl of cDNA using the QuantiTect SYBR Green PCR Kit (Qiagen), and specific primers (**Table 1**). A LightCycler 96 apparatus (Roche) was used for sample analysis. Samples were denatured for 15 min at 95°C, then DNA was amplified with 40 cycles at 95°C for 30 s and 60°C for 30 s. The final step was followed by cooling at 40°C for 30 s. *GAPDH*, *B2M* and *GUSB* were used as endogenous control for normalization. Relative quantification was realized using the 2^-ΔΔ^ *^CT^* method (41).

**Table 1:** List of primers used for the quantification of human gene expression by RT-qPCR.

Human gene	Forward primer (5’ to 3’)	Reverse Primer (5’ to 3’)
***GUSB***	ATGCCATCGTGTGGGTGAAT	TGGCGATAGTGATTCGGAGC
***B2M***	AAGTGGGATCGAGACATGTAAGC	GGAATTCATCCAATCCAAATGCG
***GADPH***	CACCATCTTCCAGGAGCGAG	GAGATGATGACCCTTTTGGC
***CXCL10***	GTGGCATTCAAGGAGTACCTC	TGATGGCCTTCGATTCTGGATT
***DDX58***	GACCCTGGACCCTACCTACA	CTCCATTGGGCCCTTGTTGT
***IFI16***	TAGAAGTGCCAGCGTAACTCC	TGATTGTGGTCAGTCGTCCAT
***IFH1***	TCACAAGTTGATGGTCCTCAAGT	CTGATGAGTTATTCTCCATGCCC
***IRF1***	ATGCCCATCACTCGGATGC	CCCTGCTTTGTATCGGCCTG
***IRF7***	CCCAGCAGGTAGCATTCCC	GCAGCAGTTCCTCCGTGTAG
***ISG15***	CACCGTGTTCATGAATCTGC	CTTTATTTCCGGCCCTTGAT
***MX2***	CAGAGGCAGCGGAATCGTAA	TGAAGCTCTAGCTCGGTGTTC
***OAS2***	ACGTGACATCCTCGATAAAACTG	GAACCCATCAAGGGACTTCTG
***RSAD2***	GCAACTACAAATGCGGCTTC	GGCTCTCCACCTGAAAAGTTG
***STAT1***	ATCAGGCTCAGTCGGGGAATA	TGGTCTCGTGTTCTCTGTTCT
***STAT2***	CTGCTAGGCCGATTAACTACCC	TCTGATGCAGGCTTTTTGCTG

### 2.10 Immunoblot analysis

Cells were washed 3 times in cold PBS and lysed in RIPA buffer (25 mM Tris HCl pH 8.8, 50 mM NaCl, 0.5% Nonidet P-40 and 0.1% sodium dodecyl sulphate supplemented with cocktails of protease inhibitors). After centrifugation at 16,000 g for 20 min at 4°C, supernatant was collected and total protein concentration determined by Micro BCA™ Protein assay (Thermo Scientific, Pierce). Equal amount of protein was heated at 95°C in the presence of β-mercaptoethanol and separated by SDS-PAGE on a Mini-PROTEAN TGX Stain Free Gel (Bio-Rad). Samples were then transferred on a 0.2μm nitrocellulose membrane (Trans-Blot Turbo Transfer Pack, Bio-Rad) using a Trans-Blot Turbo Transfer system (Bio-Rad). Membranes were blocked with PBS containing 5% dry milk and 0.05% Tween-20. The membrane was then incubated with the required dilution of specific antibodies raised against ORF2 (mouse, 1/2000 dilution, clone 1E6, Merck Millipore), actin (mouse, 1/2000 dilution, clone M2, Sigma), DDX58 (mouse, 1/1000 dilution, clone Alme-1, AdipoGen Life Sciences), IFIH1 (rabbit, 1/1000 dilution, AT113, ALX-210-935, Enzo Life Sciences), or IRF1 (rabbit, 1/1000 dilution, VPA00801, Bio-Rad). After several washes, a second incubation was performed in horseradish peroxidase-conjugated goat anti-rabbit or anti-mouse secondary antibodies (1/5000, ThermoFisher Scientific) or StarBright Blue 700 goat anti-rabbit IgG (1/5000, Bio-rad). Target proteins were detected using a chemiluminescent detection system (Clarity Western ECL Substrate, Bio-Rad) and a Chemidoc Imaging system (Bio-Rad). Band intensity was measured using the Image Lab software (Bio-Rad).

### 2.11 CXCL10 enzyme-linked immunosorbent assay

The Human sIP-10/CXCL10 solid-phase sandwich ELISA (Invitrogen) was used to quantify human CXCL10 in the supernatants of HepaRG cells according to the supplier’s protocol.

### 2.12 Immunostaining and fluorescent microscopy

Cells were seeded onto a 15µ- 24-well IBIDI plate (Clinisciences). After infection, cells were fixed with 4% paraformaldehyde in PBS. Cells were then permeabilized with 0.2% Triton X-100 in PBS and incubated in blocking buffer (0.5% BSA in PBS). Anti-ORF2 antibody (mouse, 1/500 dilution, 1E6, Millipore) was then added for 1h at room temperature. Cells were then washed several times in PBS and incubated with a DyLight™ 488 anti-mouse secondary antibody (Thermo Scientific). After several washes in PBS, cell nuclei were stained with 4,6- diamidine-2-phenylindole dihydrochloride (DAPI) (Sigma-Aldrich). Microscopy was carried out with an Axio observer Z1 fluorescent microscope (Zeiss) and images were acquired using the Zen 2012 software.

### 2.13 Statistical analyses

For the PCR array data, the p-values were determined by the RT^2^ Profiler PCR Arrays & Assays Data Analysis software (Qiagen) and calculations were based on a Student’s t-test of the replicate 2^–ΔCT^. For the qPCR and CXCL10 ELISA experiments, an unpaired t-test with Welch’s correction was used to analyze the data using GraphPad Prism version 9.2.0. The statistical analysis on the qPCR data was performed using the ΔCt values.

## 3 Results

### 3.1 Identification of differentially-expressed ISGs in HEV-infected swine livers

**Figure 1:**
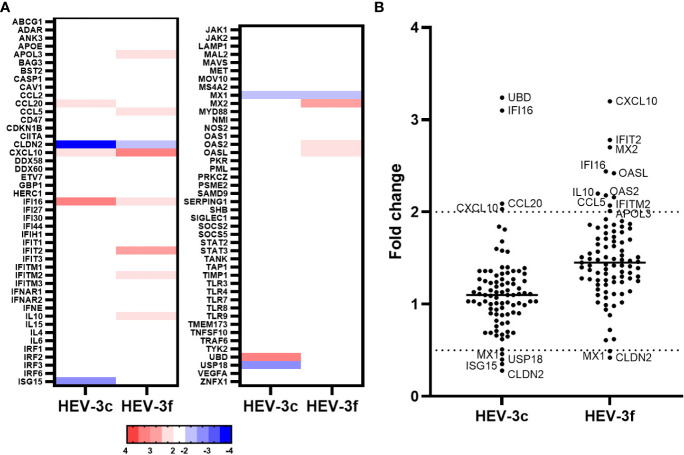
Analysis of the expression of IFN-regulated genes in liver cells from pigs infected with HEV-3c and HEV-3f. Liver samples from 2 controls, 3 HEV-3c and 3 HEV-3f infected pigs were collected 8 days post-infection (peak excretion) and were analyzed using a RT^2^ Profiler PCR array designed to study the expression of 84 genes involved in the IFN response. **(A)** Heat map showing the differential expression (fold regulation) of the 84 analyzed swine genes. Up- and down-regulated genes are colored in red and blue, respectively. **(B)** Graph representing the fold changes obtained for the different studied genes.

First, we wanted to determine which ISGs are differentially expressed during transient HEV infection in swine. Liver samples from pigs infected intravenously with HEV-3c or HEV-3f (10^6^ copies of HEV RNA) or mock-infected with PBS were collected at the peak of excretion (8 days p.i.). Similar kinetics of HEV fecal excretion were observed for both subtypes during infection (37). First, HEV RNA was quantified in these liver samples by RT-qPCR to evaluate HEV infection (**Supplementary Table 1A**). A PCR array (Qiagen) was then performed to determine the expression of 89 porcine IFN-related genes (**Figure 1**; **Supplementary Table 2**). Four genes were found to be up-regulated (*CCL20, CXCL10, IFI16* and *UBD*) and 4 down-regulated (*CLDN2, ISG15, MX1* and *USP18*) in the liver cells of pigs infected with HEV-3c. The expression of a higher number of ISGs (*APOL3, CCL5, CXCL10, IFI16, IFIT2, IFITM2, IL10, MX2, OASL* and *OAS2*) were up-regulated in pigs infected with HEV-3f and 2 were down-regulated (*CLDN2* and *MX1*) (**Figure 1**). The expression of 2 genes was found to be up-regulated (*CXCL10* and *IFI16*) or down-regulated (*CLDN2* and *MX1*) in both HEV-3c and HEV-3f infected animals.

### 3.2 HepaRG cells are able to respond to IFN

**Figure 2:**
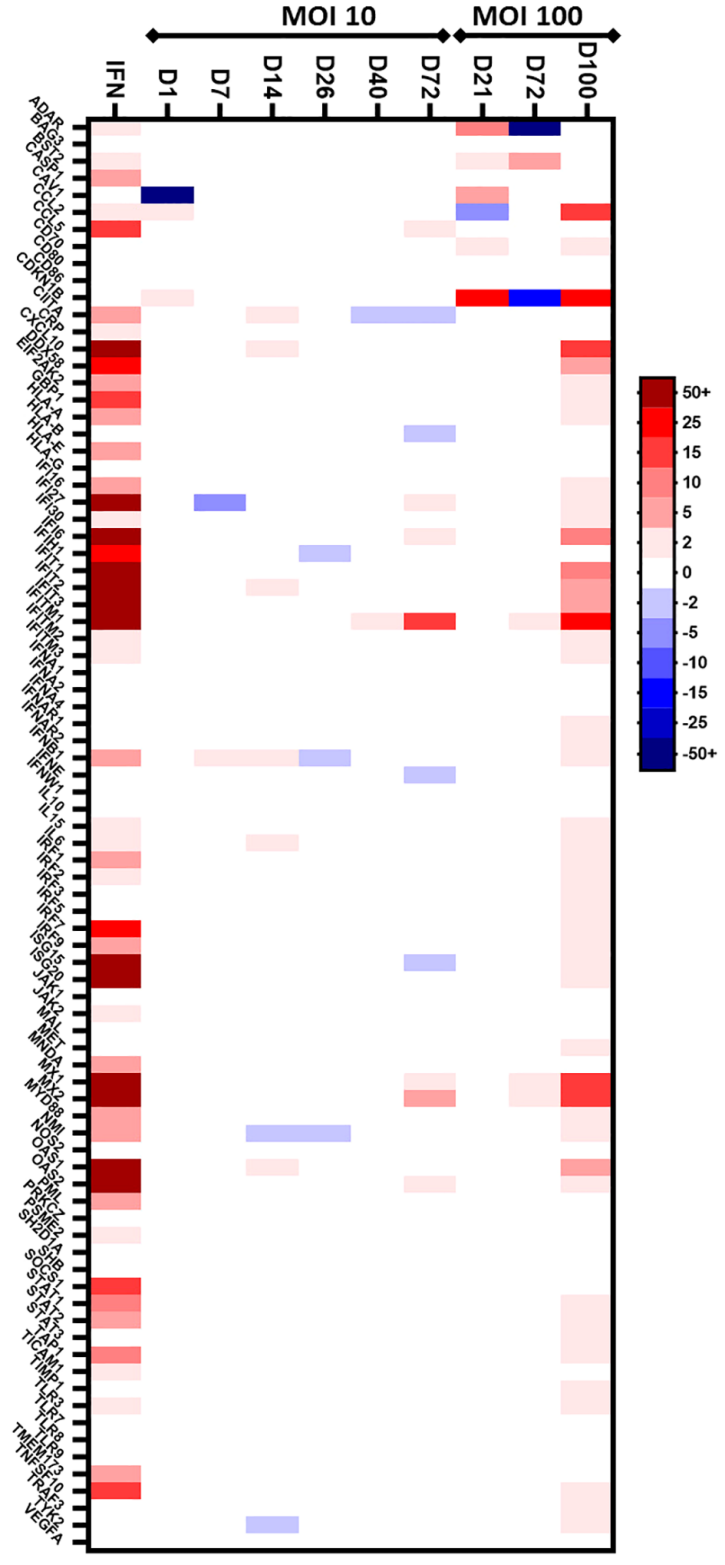
Preliminary screen to analyze the expression of IFN-regulated genes in HepaRG cells infected with HEV-3f. HepaRG cells infected with HEV-3f at a MOI of 10 or 100 GE/cell and their matched non-infected controls were analyzed at different days (D) after infection using the Human Type I Interferon Response RT² Profiler PCR Array (Qiagen). The heat map shows the differential expression of 84 analyzed human genes. The color bar represents gene expression level where up- and down-regulated genes are colored in red and blue, respectively. For MOI 10, 1 sample per time point was analyzed. For MOI 100, 1 sample containing pooled RNA from triplicate samples was analyzed.

In a second part, we aimed to identify IFN-regulated genes that are differentially expressed during chronic HEV-3f infection in human hepatic cells, the main site of HEV replication, using HepaRG cells. First, we validated that HepaRG are able to respond to IFN-I using a PCR array (Human Type I Interferon Response RT² Profiler PCR Array, Qiagen) to study the expression of 8 IFN and receptor genes and 76 genes involved in IFN signaling. After overnight treatment with IFN-β, the expression of 52 out of the 76 genes tested was up-regulated (**Figure 2**; **Supplementary Table 3**). This result confirms the ability of HepaRG to respond to IFN-I treatment and a conserved functional integrity for this pathway.

### 3.3 HEV triggers an IFN response during chronic infection in HepaRG

**Figure 3:**
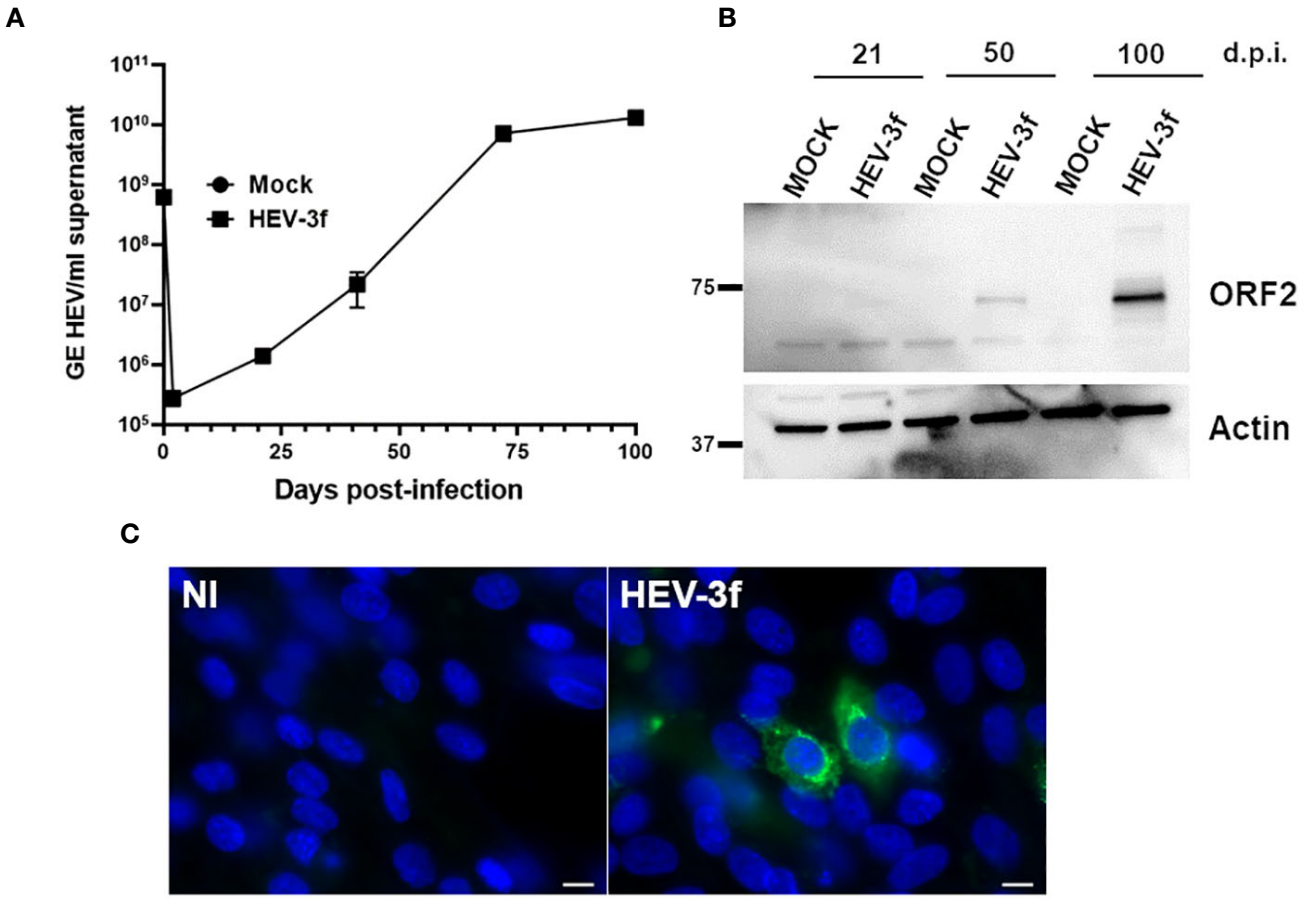
Infection of HepaRG with HEV-3f. **(A)** HEV RNA (GE/ml supernatant) in the supernatant of HepaRG cells infected at MOI 100 (GE/cells) with HEV-3f for up to 100 days. Means from triplicate samples ± SD are shown. **(B)** Detection of HEV capsid (ORF2) in the lysates of HepaRG cells infected with HEV-3 at a MOI of 100 (GE/cells) at several days post-infection (d.p.i.). **(C)** Detection of HEV ORF2 by fluorescence microscopy after staining with an anti-ORF2 antibody in HepaRG cells not-infected (NI) or infected with HEV-3f at MOI 100 GE/cells for 100 days. Nuclei were stained using DAPI. Scale bars: 10 μM.

Next, a preliminary screen was performed using whole-cell RNA extracts prepared from HepaRG mock-infected or infected cells to determine whether HEV was able to trigger an IFN response. Different MOI (10 and 100 GE/cell) and time points (D+7, D+14, D+26, D+40, D+72 and D+100) were analyzed using the PCR array (**Figure 2**; **Supplementary Table 3**). At a MOI of 10, no change in the expression of the genes coding for IFN-α (subtypes 1, 2 and 3), IFN-ϵ and IFN-ω was detected and a slight up-regulation of the expression of the gene coding for IFN-β was detected only after 7 and 14 days in HEV-infected HepaRG. Moreover, the expression of only a few ISGs was modulated after infection with HEV for up to 72 days. More important changes in ISG expression were observed later during HEV infection (D+100) when a MOI of 100 was used. To assess that the virus was replicating, we quantified the HEV RNA genome present in the supernatant of infected HepaRG cells (**Figure 3A**) and intracellularly (**Supplementary Table 1B**). Maximal amount of HEV RNA was detected 72 and 100 days after infection at MOI 100. In agreement with this result, the HEV capsid, ORF2, was not detected by immunoblot at 21 days post-infection in infected HepaRG cells but only after 50 days after infection and at higher level at 100 days post-infection (**Figure 3B**). These results suggest that HEV replication in HepaRG is slow and that an IFN response is detectable only when maximal replication is reached. Moreover, only a low proportion of cells were found to be infected by immunofluorescence microscopy after 100 days of infection (**Figure 3C**).

**Figure 4:**
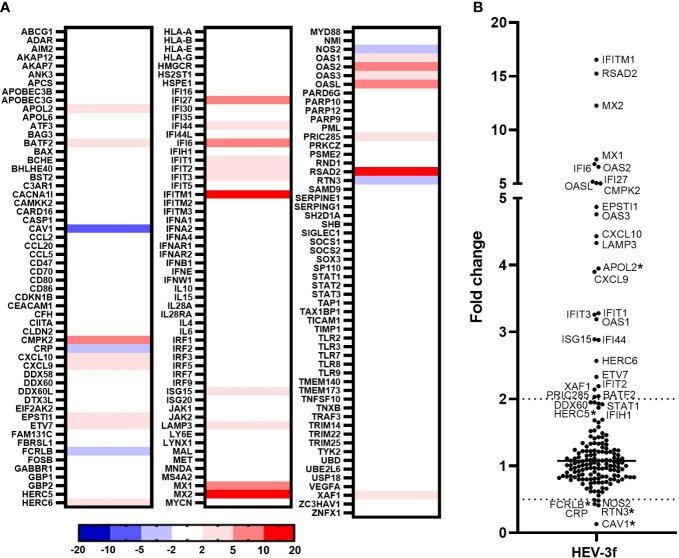
Analysis of the expression of IFN-regulated genes in HepaRG cells infected with HEV-3f. HepaRG cells infected with HEV-3f at a MOI of 100 GE/ml for 100 days and their matched non-infected controls were analyzed using RT^2^ Profiler PCR arrays designed to study the expression of 168 genes involved in the IFN response. **(A)** Heat map showing the differential expression (fold regulation) of the 168 analyzed human genes. Up- and down-regulated genes are colored in red and blue, respectively. Fold regulations were calculated by the RT^2^ Profiler PCR Arrays & Assays Data Analysis software (Qiagen) using average ΔCT values obtained from 2 independent experiments performed in triplicates. **(B)** Graph representing the fold changes obtained for the different studied genes. Fold changes with a p-value ≥ 0.05 are indicated by an asterisk (*).

The expression level of a higher number of IFN-regulated genes was then determined in HepaRG infected at a MOI of 100 for 100 days as these conditions were more favorable for the detection of differentially expressed ISGs. Quantification of HEV RNA in these samples is shown in **Supplementary Table 1C**. A PCR array was designed to study the expression of additional ISGs that were found to be differentially expressed in hepatic cells following IFN stimulation according to the interferome v2.01 database (40). The expression of a total of 168 ISGs were analyzed, among which 74 are orthologues of genes analyzed in the swine PCR arrays described above. The expression of 25 ISGs (*BATF2, CMPK2, CXCL9, CXCL10, EPSTI1, ETV7, HERC6, IFI27, IFI44, IFI6, IFIT1, IFIT2, IFIT3, IFITM1, ISG15, LAMP3, MX1, MX2, OAS1, OAS2, OAS3, OASL, PRIC285, RSAD2, XAF1*) was found to be significantly up-regulated and 2 IFN-regulated genes (*CRP* and *NOS2*) down-regulated in HepaRG cells infected with HEV-3f out of the 168 genes analyzed (**Figure 4**; **Supplementary Table 4**). *APOL2* was also found to be up-regulated and *CAV1*, *FCRLB* and *RTN3* down-regulated but not significantly (p ≥ 0.05). In addition, the expression of *DDX60*, *STAT1*, *IFIH1* expression was also up-regulated significantly but with a fold change slightly lower than 2 (fold change >1.9 with p ≤ 0.05) (**Supplementary Table 4**). No change in the expression of the genes coding for IFN-α (1, 2 and 3), IFN-β and IFN-λ2 was detected.

### 3.4 Validation of the PCR array data by additional RT-qPCR, immunoblotting and ELISA

**Figure 5:**
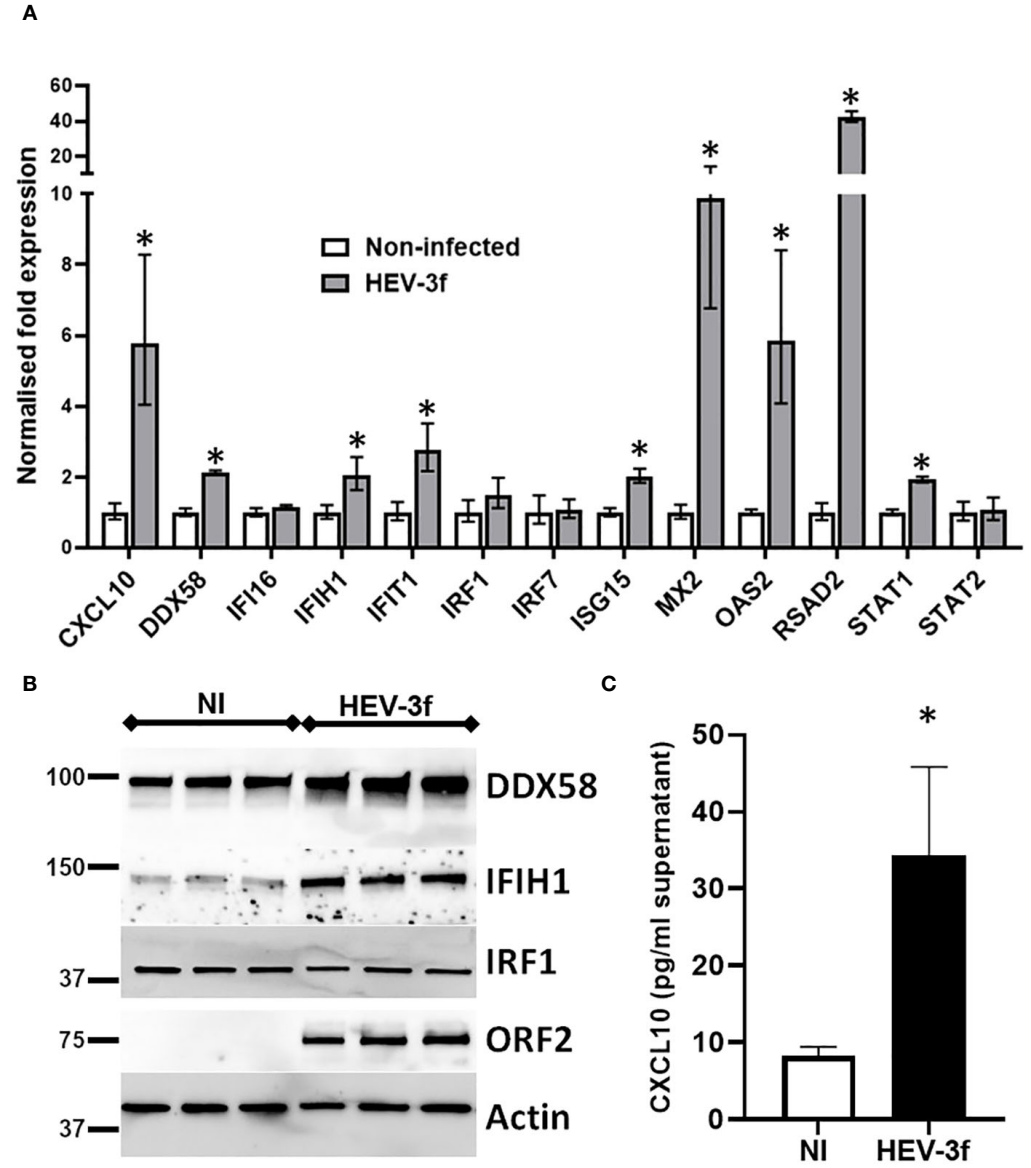
Validation of the PCR array data. **(A)** HepaRG cells were infected or not with HEV-3f for 100 days at MOI 100 and the expression of selected genes shown by PCR array to be up-regulated or unchanged was analyzed by RT-qPCR. The results shown are the geometric means ± SD from 3 to 4 replicate samples and are representative of 2 independent experiments. *GUSB*, *B2M* and *GAPDH* were used as reference genes. Unpaired t-test with Welch’s correction Mann-Whitney test, *: p<0.05 **(B)** Immunoblot showing the expression of the protein encoded by *IFIH1*, *DDX58* and *IRF1* in HepaRG cells infected with HEV-3f or not (NI) for 100 days at MOI 100 for 3 replicate samples. Representative blots from 2 independent experiments are shown. ORF2 and actin protein levels were also detected as control of infection and loading, respectively. **(C)** Detection of CXCL10 in the supernatant of non-infected HepaRG (NI) or HepaRG cells infected with HEV-3f for 100 days at MOI 100 (GE/cell) by ELISA. The results shown are the means ± SD from 4 replicate samples and are representative of 2 independent experiments. Unpaired t-test with Welch’s correction, *: p<0.05.

Additional RT-qPCR tests were then performed to confirm the data obtained with the PCR arrays for several genes which expression was found to be unchanged (*DDX58, IFI16, IRF1, IRF7* and *STAT2*), significantly up-regulated with fold change >2 (*CXCL10*, *IFIT1*, *ISG15*, *MX2*, *OAS2* and *RSAD2*) or between 1.9 and 2 (*IFIH1* and *STAT1*) (**Figure 5A**). Similar results were obtained by RT-qPCR except for the mRNA level of *DDX58* that was found to be significantly up-regulated by RT-qPCR but unchanged in the PCR array (fold change 1.4) after HEV-3f infection. This up-regulation of *DDX58* expression was confirmed by immunoblot at the protein level (**Figure 5B**). Immunoblot analysis also showed that the expression of IFIH1 is increased and the one of IRF1 unchanged after HEV infection, corroborating the results obtained by PCR array and RT-qPCR. Increased level of CXCL10 was also detected by ELISA in supernatant of HepaRG cells infected with HEV-3 (**Figure 5C**), confirming the up-regulation of *CXCL10* detected by PCR array (**Figure 4**).

## 4 Discussion

In the present study, we have determined the expression of up to 168 genes involved in the IFN antiviral response in HEV-infected swine livers and human HepaRG cells using customized PCR arrays.

**Figure 6:**
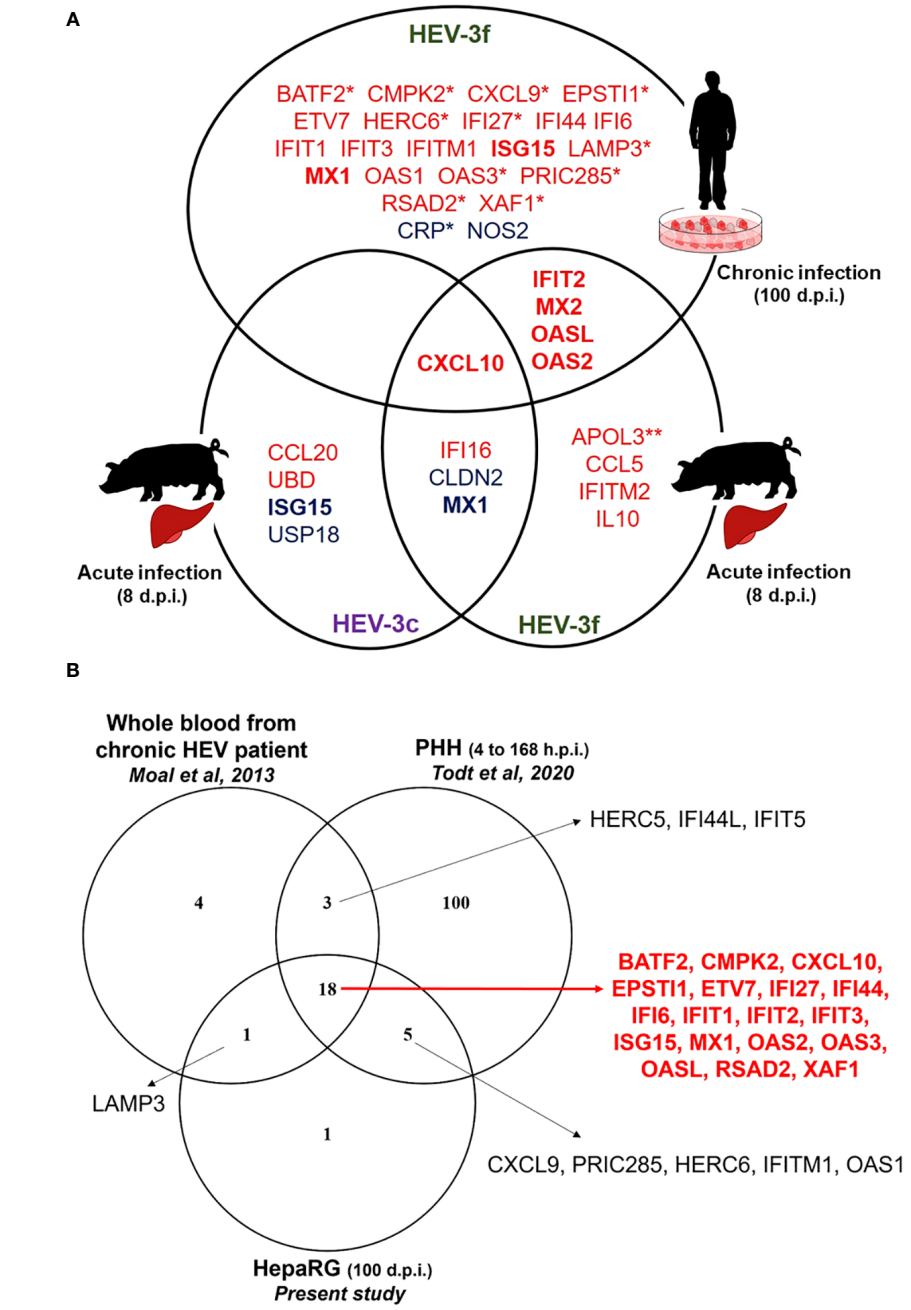
IFN-regulated genes differentially expressed during HEV infection in HepaRG cells and pig liver tissues **(A)** Venn diagram representing the genes shown to be differentially expressed during HEV-3 infection in human HepaRG cells and pig liver cells in this study. Up-regulated and down-regulated genes are shown in red and in blue, respectively. *: only tested in HepaRG. **: only tested in pig liver. d.p.i.: days post-infection. **(B)** Venn diagram representing IFN-regulated genes shown to be up-regulated during HEV-3 infection in human cells in different studies. The genes shown to be up-regulated in the three studies are indicated in red. PHH: primary human hepatocyte; h.p.i.: hours post-infection; d.p.i.: days post-infection.

In the liver of pigs experimentally infected with HEV-3, 12 ISGs with diverse functions were found to be up-regulated and 4 down-regulated at the peak of excretion (8 days post-infection) (**Figure 6A**; **Supplementary Table 5**). Two subtypes of HEV-3 were used for the infection, HEV-3c and HEV-3f. These subtypes are the most common in Europe. They belong to 2 different clades and share 82% identity in their nucleotide sequences (5). Only 2 of the 12 up-regulated ISGs (*CXCL10* and *IFI16*) and 2 of the 4 down-regulated (*CLDN2* and *MX1*) were common between HEV-3c and HEV-3f infection, suggesting that the IFN response might differ depending on the HEV subtype involved in infected swine. Higher expression of the gene coding for CXCL10 (or IP-10 for interferon gamma-induced protein 10) after HEV infection has already been reported in hepatocytes and enterocytes *in vitro*, in liver biopsies from rhesus macaques and chimpanzees, in human liver chimeric mice and in patients with chronic hepatitis E (**Figure 6B**; **Supplementary Table 6**) (44–50). Higher level of CXCL10 has also been detected in serum and whole blood samples from patients with acute hepatitis E (HEV-1 and HEV-3) (51, 52) and has been linked to more severe symptoms in patients infected with HEV-3 (52). CXCL10 is a chemokine that is involved in the recruitment of T cells and plays a role in the pathogenesis associated with several viral infections (53). Interestingly, higher levels of CXCL10 have also been reported with other hepatotropic viruses such as hepatitis B virus (HBV) and HCV and it was suggested that this chemokine could be used as a biomarker predicting liver injury and phases of infection in the context of HBV and liver fibrosis in the context of HCV (54–56). Further investigations are needed to determine whether increased level of CXCL10 during hepatitis E infection can be used as a marker of disease severity. The expression of *IFI16* was also found to be up-regulated in the liver of pigs infected with both subtypes of HEV-3 but not in chronically infected HepaRG cells. IFI16 is a cytosolic DNA sensor involved in the recognition of DNA viruses but more recent studies have shown that this host protein is also involved in the sensing and restriction of several RNA viruses including influenza A virus (IAV), porcine reproductive and respiratory syndrome virus 2 and chikungunya virus (CHIKV) (57). It was shown that IFI16 interacts with IAV and CHIKV genomic RNA and positively regulates retinoic acid-inducible gene I (RIG-I) signaling during IAV infection (58–60). However, the mechanisms involved in the antiviral effect of IFI16 on RNA viruses remain to be fully identified. In the future, it would be interesting to investigate whether this ISG plays a role in the control of HEV infection, particularly in the swine host. The other ISGs whose expression was found to be up-regulated in swine differ depending on the subtypes used for the infection. Interestingly, the expression of *MX2*, *IFIT2*, *OASL* and *OAS2* was up-regulated after infection with HEV-3f in both pig livers and HepaRG cells but not in pig livers infected with HEV-3c. Moreover, *ISG15* and *USP18* mRNA levels were down-regulated (HEV-3c) or unaffected (HEV-3f) in pig livers in this study but were shown to be up-regulated in the liver of pigs infected with HEV-3a in another study (61). These results suggest then that the expression of some ISGs might be differentially modulated depending on the subtypes or strains of HEV-3 involved. Interestingly, ISG15 has been shown to harbor immunomodulatory function by negatively regulating IFN signaling in the context of HEV infection (61, 62). Differences in the expression of these ISGs could be linked to the differences in the severity of infection that exist between HEV-3 subtypes, HEV-3c being less likely to lead to hospitalization and death than HEV-3f (63, 64). The antiviral response might also be influenced by the genetic background of the pigs used for the infection. The expression of some IFN-regulated genes was also shown to be down-regulated in HEV-infected swine liver tissues (*CLDN2*, *MX1*, *ISG15* and *USP18*). Interestingly, the expression of *MX1* was down-regulated (HEV-3c and HEV-3f) and the expression of *ISG15* unaffected (HEV-3f) or down-regulated (HEV-3c) in pig liver tissues whereas their expression was up-regulated in persistently infected HepaRG cells or other human models of HEV infections (**Figure 6B**) (45, 47, 65). It will be interesting to investigate further whether these down-regulations occur specifically in swine or with the strains used for the infection and whether these differences are relevant to the pathogenesis of HEV. Differences in the expression profile of *IFN* and IFN-responsive genes have already been reported between HEV-1 and HEV-3 suggesting that the host response controlling the infection differs depending on the genotype or strain of HEV involved (20). In the liver of infected rhesus macaques, multiple IFN response genes were found to be down-regulated early after infection with HEV-1 but up-regulated with HEV-3 (46).

In HepaRG cells persistently infected with HEV-3f, no increase in the expression of several genes coding for IFN (different subtypes of IFN-α, IFN-β and IFN-λ2) was detected and the expression of 25 ISGs was found to be significantly up-regulated (fold change ≥2) (**Figure 6A**). As the functional integrity of HepaRG to respond to IFN was confirmed at the time of infection, it suggests that a weak IFN response is triggered by HEV-3f in this model or that IFN responsiveness of the HepaRG has changed over long period of infection. This weak antiviral response might also be caused by the low proportion of cells infected. As HEV is able to counteract the IFN system pathways via different mechanisms (20), it is also possible that these inhibitory functions are efficient to block IFN signaling and response in HepaRG cells, especially at early stages of infection when lower levels of viral RNA are present. Moreover, these results indicate that HEV-3f is able to persist in infected HepaRG cells albeit up-regulation of the expression of these 25 ISGs, possibly by circumventing their effects or because chronically-infected cells have become refractory to the effect of a prolonged IFN response. These 25 ISGs include genes coding for cytokines, transcription factors, proteins involved in the ubiquitin and ISGylation pathways as well as viral RNA/DNA sensors and antiviral proteins interfering with different steps of the viral life cycle (**Supplementary Table 5**). The ability of HEV to interfere with IFN signaling and response (18, 20, 28) and the absence of production of high level of IFN in infected HepaRG cells could explain why the virus can persistently infect these cells. Several transcriptomic studies have already reported the up-regulation of several ISGs in the context of HEV-1 and HEV-3 infection (**Supplementary Table 6**) (44–47, 65, 66). Eighteen of the 25 up-regulated genes identified here, in persistently infected HepaRG cells, were also found in studies realized in patients with chronic HEV infection and in infected primary human hepatocytes (PHH) and might represent core ISGs expressed during HEV in human cells and host (**Figure 6B**; **Supplementary Table 6**). Some other core ISGs might exist but were not identified as different models and technologies were used in these studies and the whole transcriptome was not determined in all of them. Moreover, in this study, we found an increase in the level of *DDX58* mRNA by RT-qPCR but this increase was not significant using the PCR array suggesting that this technique might not have allowed to detect the modulation of expression of some ISGs. Interestingly, 19 out of the 26 ISGs found to be up-regulated in the blood of patients with chronic HEV (45) were similar to the ones found in this study in persistently infected HepaRG cells (**Figure 6B**; **Table S6**). This strengthens the relevance of the HepaRG system as model to study the innate immune response in the context of chronic hepatitis E infection (34). This is of particular interest as it is difficult to have access to liver biopsies from patients with chronic hepatitis E and PHH are less available, display variability between donors and can be difficult to culture over long periods of time. Moreover, it would be interesting to develop a model of HEV infection in swine hepatic cells. However, no hepatic cell lines are commercially available yet and, like PHH, access to primary hepatocytes from pigs is limited.

It is essential now to determine whether the ISGs, whose expression was found to be modulated during HEV infection in the present study (as summarized in **Figure 6A**), have a direct or indirect antiviral activity against HEV and play a role in the control or persistence of HEV infection. Some of the ISGs identified are sensors or adaptors of the IFN signaling pathways (**Supplementary Table 5**) and may play an indirect role in amplifying or regulating IFN secretion and response. In contrast, some of these ISGs have been shown to act as direct effectors of the IFN response (**Supplementary Table 5**) and could have direct antiviral activity against HEV. It has already been shown that IFIT1 has antiviral activity against HEV by preventing HEV RNA translation (33) and that its expression is up-regulated upon HEV infection in human cells (**Figure 6B**; **Table S6**). Interestingly, HEV RNA-dependent RNA polymerase is able to bind to IFIT1 and interferes with its antiviral activity, thus suggesting that IFIT1 plays an important role in the control of HEV infection (33). In this study, we have also showed that *IFIH1* and *DDX58* expression was up-regulated in HEV-3f persistently infected HepaRG at the mRNA and protein level by qPCR and immunoblot analysis. Melanoma differentiation-associated protein 5 (MDA5, encoded by *IFIH1*) and RIG-I (encoded by *DDX58*) are both viral RNA sensors that were already shown to inhibit HEV infection (67, 68). Studies have also reported that two other ISGs, IRF1 and GBP1, are able to interfere with HEV infection (69, 70). No modulation of the expression of these 2 genes was detected here in HEV infected pig liver tissues or in persistently infected HepaRG cells. Hence, in the future, it would be interesting to investigate whether HEV has evolved strategies to prevent up-regulation of the genes encoding MDA5, RIG-I, IRF1 and GBP1. These 4 proteins share 78% to 90% sequence homology between the human and porcine species and could interfere with HEV-3 infection in both species. Some ISGs could also have proviral properties and play a role in the pathogenesis of HEV infection. As described above, ISG15 is of particular interest for future studies as differences in its expression profile were detected in the different models studied here.

In conclusion, this study has allowed us to identify ISGs whose expression is modulated during acute HEV infection in the liver tissues of infected pigs and during chronic HEV infection in human hepatic cells. This is the first *in vivo* study to assess the expression of multiple ISGs in pigs infected with two strains of HEV-3. The use of this model of HEV infection is of particular interest as the virus causes acute infection in swine and no apparent symptoms. It could then provide useful clues on the effectors of the antiviral response that are efficient to control the virus. As *in vivo* and *in vitro* models from different species were used in this study, it is not possible to directly compare the ISGs profiles identified. However, together with the literature already available (**Supplementary Table 6**), it provides useful information on potential ISGs that might play a role in the host antiviral response and in the control or persistence of viral replication. We are now investigating the ability of these ISGs of interest to modulate HEV infection to correlate their expression profiles with putative pro- or anti-viral functions. We will also investigate whether the anti-HEV antiviral activity of these ISGs varies depending on the species involved (human vs porcine). This will provide a better understanding of the effector functions of the IFN response activated during HEV infection and how this response influences pathogenesis and inter-species transmission of HEV. By identifying cellular antiviral molecules able to inhibit HEV and their mode of action, such knowledge will also greatly contribute to the identification of new antiviral targets. This is particularly important for the treatment of chronic hepatitis E in immunosuppressed patients, where efficient antiviral therapies with low secondary effects are needed.

## Data availability statement

The PCR array datasets generated for this study have been deposited in the GEO (Gene Expression Omnibus) data repository under GEO superseries GSE243864 containing GEO accession numbers GSE243855, GSE243856, GSE243860 and GSE243862.

## Ethics statement

Ethical approval was not required for the studies on humans in accordance with the local legislation and institutional requirements because only commercially available established cell lines were used. The animal study was approved by Ethics committee (ComEth number 12-043) of the National Veterinary School of Alfort, the French Agency for Food, Environmental and Occupational Health & Safety, and University Paris 12 and has obtained formal approval (notice number 09/10/12-9). The study was conducted in accordance with the local legislation and institutional requirements.

## Author contributions

LM: Conceptualization, Formal analysis, Investigation, Methodology, Writing – review & editing. ID: Formal analysis, Investigation, Methodology, Writing – review & editing. SG: Investigation, Methodology, Writing – review & editing. MP: Investigation, Methodology, Writing – review & editing. AM: Investigation, Methodology, Writing – review & editing. NP: Conceptualization, Resources, Supervision, Writing – review & editing. VD: Conceptualization, Formal analysis, Funding acquisition, Investigation, Methodology, Project administration, Supervision, Validation, Writing – original draft, Writing – review & editing.
