# Supplementary material for: Identification of interferon-stimulated genes with modulated expression during hepatitis E virus infection in pig liver tissues and human HepaRG cells
Source: Front Immunol. 2023 Nov 20;14:1291186. doi: 10.3389/fimmu.2023.1291186 (PMC10696647; doi:10.3389/fimmu.2023.1291186)
Supplement: Supplementary file 3 [file Table_1.docx]

Supplementary Material


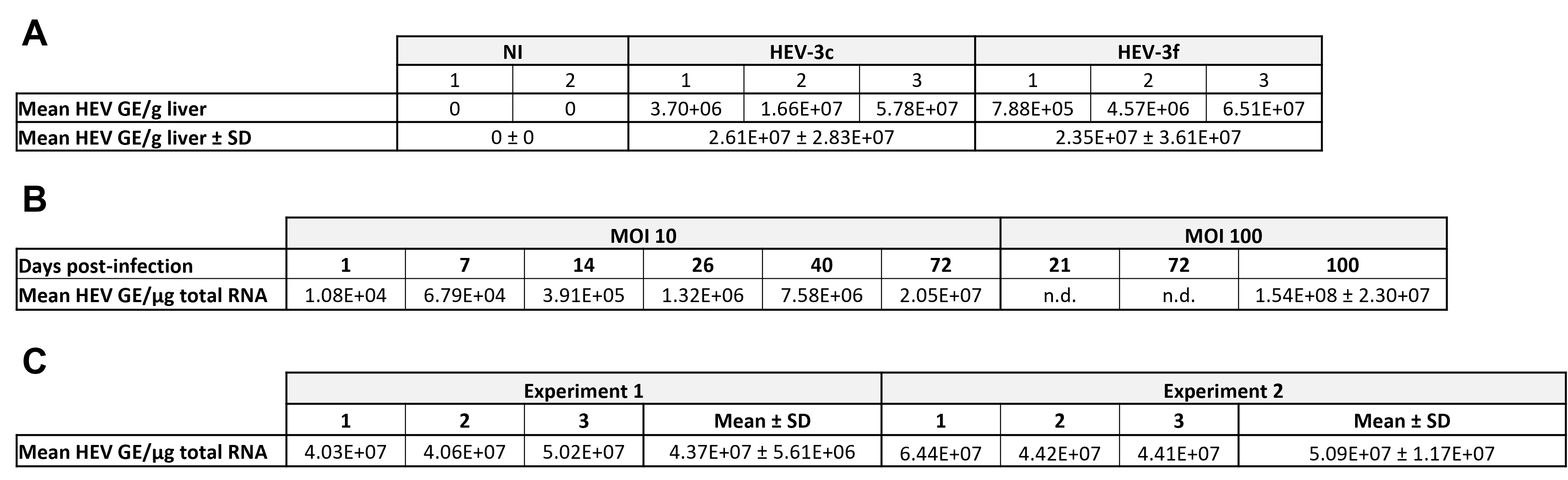


**Supplementary Table 1**: Quantification of HEV in the different samples used for the PCR arrays. (A) Quantification of HEV RNA in the pig liver samples. (B) HepaRG cells infected for different time at MOI 10 or 100 GE/cell for the preliminary screen. n.d.: not determined. (B) HepaRG infected at MOI 100 GE/cell for 100 days.


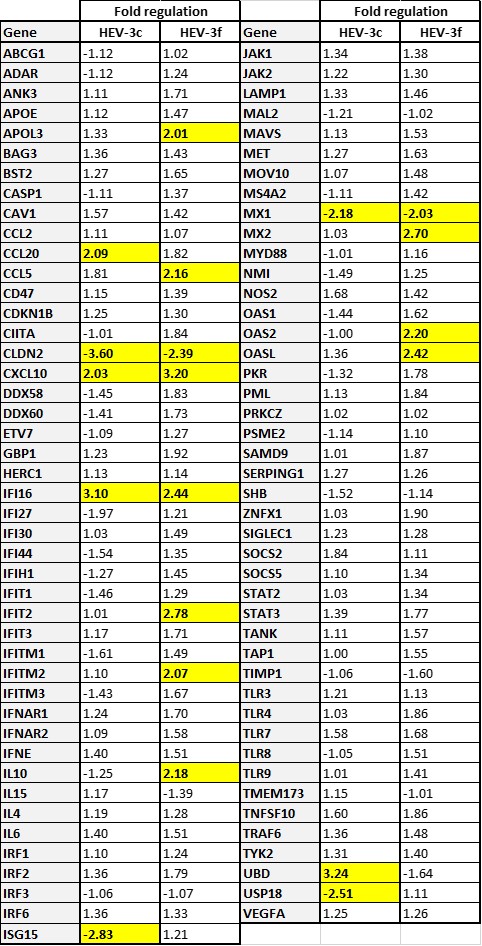


**Supplementary Table 2**: Table showing the expression level (fold regulation) of 84 genes involved in the IFN response in liver tissue samples from 3 pigs infected with HEV-3c or HEV-3f in comparison to 2 non-infected pigs as determined by RT^2^ PCR array. Genes that were shown to be up-regulated (≥ 2) or down-regulated (≤ -2) are shown in yellow.


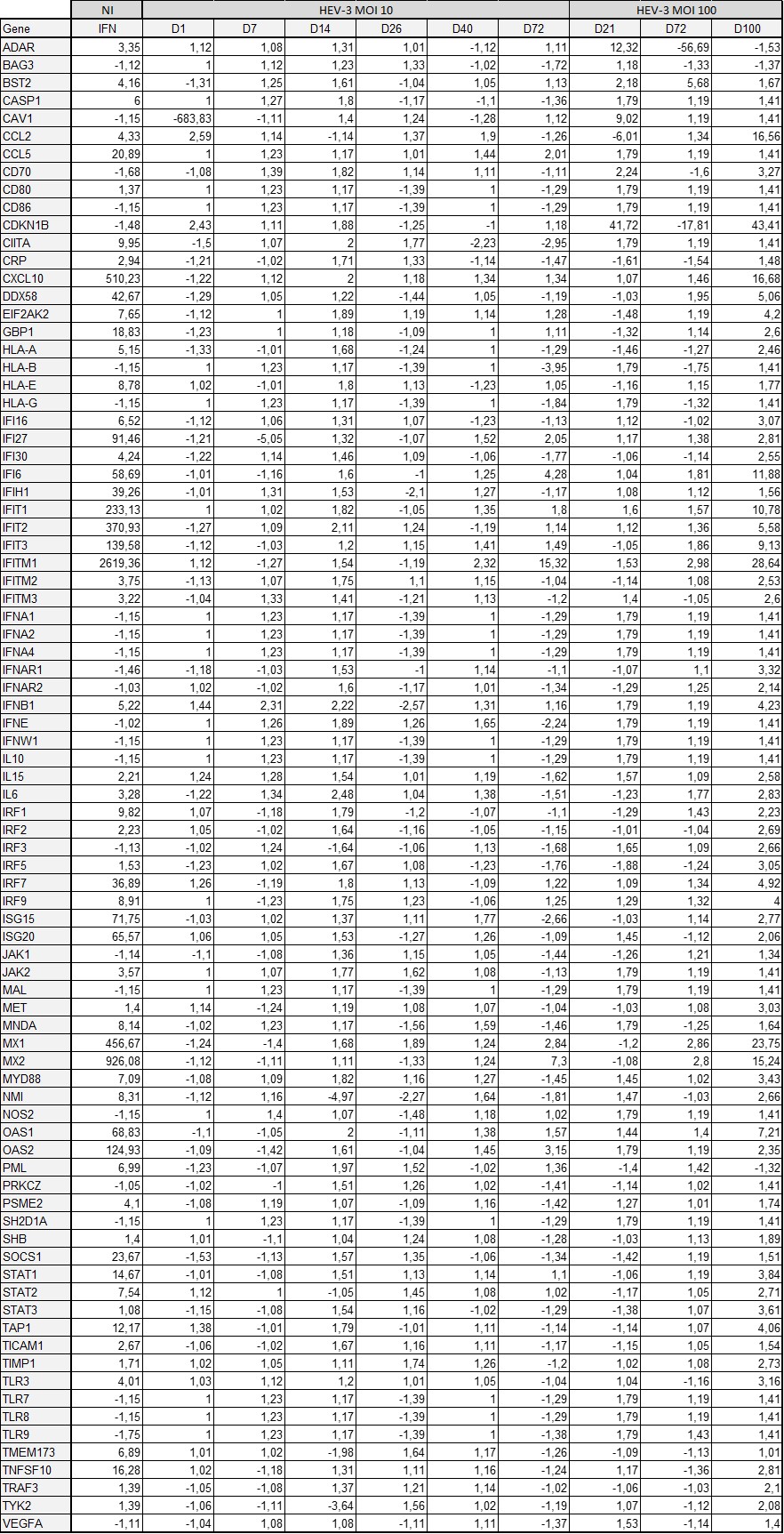


**Supplementary Table 3**: Table showing the expression level (fold regulation) of 84 genes involved in the IFN response in HepaRG cells treated with IFN or infected with HEV-3f at MOI 10 and MOI 100 in comparison to non-treated non-infected cells as determined by using RT^2^ PCR array analysis.


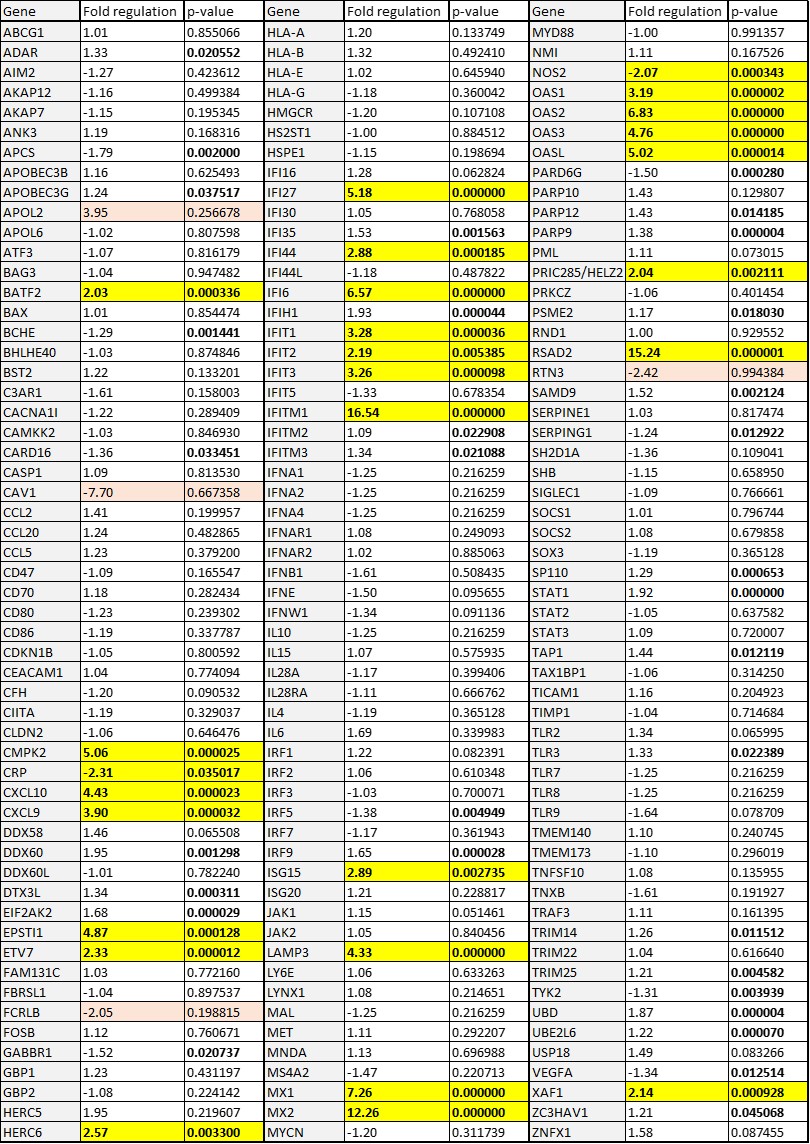


**Supplementary Table 4**: Table showing the expression level (fold regulation and p-value) of 168 genes involved in the IFN response in HepaRG cells infected with HEV-3f for 100 days at MOI 100 (GE/cell) in comparison to non-infected cells as determined by RT^2^ PCR array analysis. Results from 2 independent experiments performed in triplicates. Genes with p-values ≤ 0.05 are shown in bold. Genes that were shown to be significantly up-regulated (≥ 2) or down-regulated (≤ -2) are shown in yellow. Genes that were shown up-regulated (≥ 2) or down-regulated (≤ -2) with p-value ≥ 0.05 are shown in pink.


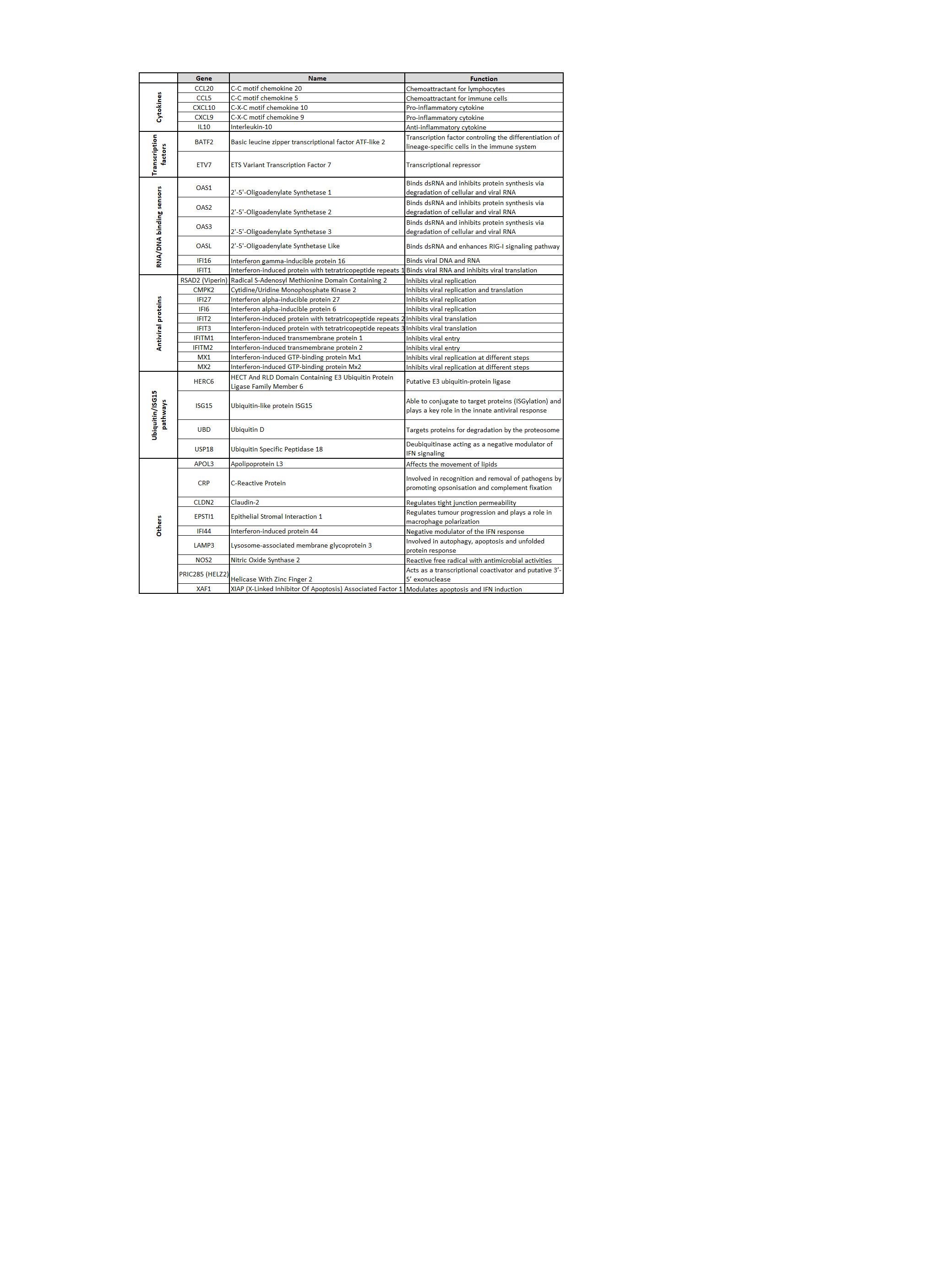


**Supplementary Table 5**: Table describing the function(s) of the genes identified as differentially expressed upon HEV infection in HepaRG cells and pig livers in this study according to (1–3).

| **Study** | **Virus genotype** | **Sample type and analysis** | **Time post-infection** | **Up-regulated genes upon HEV infection** |
| --- | --- | --- | --- | --- |
| Yu *et al*, 2010 (4) | HEV-1 (SAR-55) | Needle liver biopsies from chimpanzees inoculated intravenously and analysed by microarray (Affymetrix U133 + 2.0 human microarray chips) | First, peak and/or last positive weeks of viremia | *B2M,* ***CMPK2****,* ***CXCL10****, CXCL11,* ***CXCL9****, DDX60,* ***EPSTI1****, GABBR1, HERC5,* ***HERC6****, HLA-B, HLA-F,* ***IFI27****,* ***IFI44****, IFI44L,* ***IFI6****, IFIH1,* ***IFIT1****,* ***IFIT2****,* ***IFIT3****,* ***IFITM1****, IRF7,* ***ISG15****,* ***MX1****, NLRC5,* ***OAS1****,* ***OAS2****, PARP12, PARP9, PSMB9,* ***RSAD2****, RTP4, SAMD9, STAT1, TRIM22,* ***UBD****, UBE2L6,* ***XAF1***  **38 genes up-regulated :**  **32/38 genes tested in HepaRG**  **18/32 genes also up-regulated in HepaRG** |
| Devhare *et al*, 2013 (5) | HEV-1 | Human cells derived from human lung cancer (A549) infected *in vitro* and analysed by TaqMan low density array (95 antiviral genes tested) | 24, 48, 72 and 96 h.p.i. | *A20, ADAR, B2M,* ***CCL20****, DDX58, EIF2AK2, GBP1, GBP2,* ***IFI44****, IFIH1,* ***IFIT1****,* ***IFIT2****, IFNAR1, IFNAR2, IRF1, TLR3, IRF7, IRF9,* ***ISG15****, JAK1, JAK2,* ***MX1****,* ***OAS1****, PIAS1, PIAS2, SOCS1, SOCS2, SOCS3, STAT1, STAT2, STAT3, TLR2, TRAF3, TRAF6*  **34 genes up-regulated :**  **28/34 genes tested in HepaRG**  **6/28 genes also up-regulated in HepaRG** |
| Moal *et al*, 2013 (6) | HEV-3 | Whole blood from kidney transplant recipients with chronic hepatitis E on immunosuppressive treatment (8 chronic HEV and 8 control patients) analysed by microarray (Agilent 4X44 K Whole Human Genome) | 12 to 75 months post-infection (with a median time of 44 months) | ***BATF2****,* ***CMPK2****,* ***CXCL10****,* ***EPSTI1****,* ***ETV7****, HERC5,* ***IFI27****,* ***IFI44****, IFI44L,* ***IFI6****,* ***IFIT1****,* ***IFIT2****,* ***IFIT3****, IFIT5, IL4,* ***ISG15****,* ***LAMP3****, LY6E,* ***MX1****,* ***OAS2****,* ***OAS3****,* ***OASL****,* ***RSAD2****, SERPING1, SIGLEC1,* ***XAF1***  **26 genes up-regulated :**  **26/26 genes tested in HepaRG**  **19/26 genes also up-regulated in HepaRG** |
| Zhang *et al*, 2014 (7) | HEV-4 | Human cells derived from human hepatocellular carcinoma (PLC/PRF/5) infected *in vitro* and analysed by microarray (Affymetrix  HG-U133 Plus 2 chip) | 60 d.p.i. | ***CMPK2****,* ***IFI27****,* ***IFI6****,* ***MX1***  **4 genes up-regulated :**  **4/4 genes tested in HepaRG**  **4/4 genes also up-regulated in HepaRG** |
| Choi *et al*, 2018 (8) | HEV-1 (SAR-55) | Liver biopsies of rhesus macaques experimentally infected intravenously and analysed by specific immune gene RT^2^ profiler PCR array (Qiagen) | Early, peak, and decline phases of infection | *BCL2A1, BIRC3, CARD11, CCL11,* ***CCL20****,* ***CCL5****, CCL7, CCR2, CCR5, CCR6, CD14, CD27, CD70, CD80, CD86, CEBPB, CIITA,* ***CXCL10****, CXCR3, DDX58, EGR1, FOS, GATA3, HAVCR2,* ***IFIT1****,* ***IFIT3****, IFNG, IFNGR1,* ***IL10****, IL12B, IL15, IL18, IL1A, IL1B, IL1R1, IL1RL1, IL27, IL2RA, IL7, IL7R, IRAK2, IRF1, IRF7,* ***ISG15****, ISG20, LAG3, LAT, LBP, LYZ, MAL, MAMU-A, MAMU-B, MAMU-G,* ***MX1****,* ***MX2****, NLRP3, NOD2,* ***OAS2****, PLAU, PTPRC, REL, SAMSN1, SOCS1, SOCS2, SPP1, STAT1, STAT4, TAP1, TBX21, TLR7, TLR9, TNF*  **72 genes up-regulated :**  **26/72 genes tested in HepaRG**  **7/26 genes also up-regulated in HepaRG** |
|  | HEV-3 |  |  | *ADAR, BAG3, BIRC3, BST2, CALCOCO2, CARD11, CASP1, CAV1, CCL2,* ***CCL20****,* ***CCL5****, CCL7, CCR2, CCR5, CCR6, CD27, CD83, CD86, CDC37, CIITA,* ***CXCL10****, CXCR3, DDX58, DIABLO, EIF2AK2, GBP3, HAVCR2, HPX,* ***IFI27****, IFI30, IFI35, IFIH1,* ***IFIT1****,* ***IFIT3****,* ***IFITM1****, IFNAR1, IFNG,* ***IL10****, IL12B, IL15, IL1R1, IL1RL1, IL23A, IL2RA, IL4, IL7R, IRAK2, IRF1, IRF3, IRF5, IRF7,* ***ISG15****, ISG20, JAK1, LAG3, LBP, LYZ, MAL, MAMU-A, MAMU-B, MAMU-E, MAMU-G,* ***MX1****,* ***MX2****, MYD88, NLRP3, NMI, NOD2,* ***OAS1****,* ***OAS2****, PLAU, PML, PSME2, PTPRC, REL, RELB, SOCS1, STAT1, STAT2, STAT3, STAT4, TAP1, TBX21, TLR7, TLR8, TLR9, TNF, TNFRSF8, TNFRSF9*  **89 genes up-regulated :**  **48/89 genes tested in HepaRG**  **10/48 genes also up-regulated in HepaRG** |
| Todt *et al*, 2020 (9) | HEV-3 | Primary human hepatocytes infected with cell culture-derived HEV in vitro and analysed by RNA-sequencing | 4, 8, 12, 24, 48 and 168 h.p.i. | *ANGPTL1, APOBEC3B,* ***APOL3****,* ***BATF2****, BST2, C19orf66, C3AR1, C5AR2, CD163, CD274, CDT1,* ***CMPK2****, CX3CL1,* ***CXCL10****, CXCL11,* ***CXCL9****, DDO, DDX58, DDX60, DDX60L, DGKG, DHX58, DTL, E2F1, E2F7, EDNRA, EIF2AK2,* ***EPSTI1****,* ***ETV7****, EXOC3L1, FAM46A, FOXQ1, GALNTL6, GBP4, GBP5, GOS2, GPRIN3, GREM2, GRIP2, HAVCR2, HERC5,* ***HERC6****, HES4, HLA-F, HRASLS2, HSH2D,* ***IFI16****,* ***IFI27****, IFI35,* ***IFI44****, IFI44L,* ***IFI6****, IFIH1,* ***IFIT1****,* ***IFIT2****,* ***IFIT3****, IFIT5,* ***IFITM1****, IGF1, IGFBP5, IL1RN, IL22RA1, IRF7, IRF9,* ***ISG15****, ISG20, LAP3, LGALS9, MCHR1, MOB3C, MUC13,* ***MX1****, NLRC5, NNMT, NOD2, NPR1, NT5C3A,* ***OAS1****,* ***OAS2****,* ***OAS3****,* ***OASL****, ODF3B, OLFML1, PARP10, PARP14, PARP9, PIWIL2, PLAT, PLEKHA4, PLSCR1, PNPT1,* ***PRIC285****, PSMB9, RAB3B, RAB42, REC8, RET, RHEBL1, RMI2, RNF213,* ***RSAD2****, RTP4, SAMD9, SAMD9L, SAMDH1, SEMA6A, SLC15A3, SLC25A28, SLC9A9, SOCS1, SOCS3, SP110, STARD5, STAT1, STAT2, STEAP4, TAP1, TDRD7, TMEM217, TREX1, UBA7, UBE2L6, UNC93B1, USP18,* ***XAF1****, ZNFX1*  **126 genes up-regulated :**  **50/126 genes tested in HepaRG**  **23/50 genes also up-regulated in HepaRG** |

**Supplementary Table 6**: List of genes involved in the IFN response shown to be up-regulated after HEV infection in published transcriptomic studies. Genes were defined as ISGs as stated in the different published studies or according to the interferome database (10). ISGs found to be up-regulated in this present study after HEV infection in HepaRG and/or pig livers are indicated in red.

**References**

1. Elemam NM, Talaat IM, Maghazachi AA. CXCL10 Chemokine: A Critical Player in RNA and DNA Viral Infections. *Viruses*. (2022);14(11):2445. doi: 10.3390/v14112445

2. Schoggins JW. Interferon-Stimulated Genes: What Do They All Do? *Annu Rev Virol*. (2019) 6(1):567‑84. doi: 10.1146/annurev-virology-092818-015756

3. Stelzer G, Rosen N, Plaschkes I, Zimmerman S, Twik M, Fishilevich S, et al. The GeneCards Suite: From Gene Data Mining to Disease Genome Sequence Analyses. *Curr Protoc Bioinformatics*. (2016) 54:1.30.1-1.30.33. doi: 10.1002/cpbi.5

4. Yu C, Boon D, McDonald SL, Myers TG, Tomioka K, Nguyen H, et al. Pathogenesis of hepatitis E virus and hepatitis C virus in chimpanzees: similarities and differences. *J Virol*. (2010) 84(21):11264‑78. doi: 10.1128/JVI.01205-10

5. Devhare PB, Chatterjee SN, Arankalle VA, Lole KS. Analysis of antiviral response in human epithelial cells infected with hepatitis E virus. *PLoS ONE*. (2013) 8(5):e63793. doi: 10.1371/journal.pone.0063793

6. Moal V, Textoris J, Ben Amara A, Mehraj V, Berland Y, Colson P, et al. Chronic hepatitis E virus infection is specifically associated with an interferon-related transcriptional program. *J Infect Dis.* (2013) 207(1):125‑32. doi: 10.1093/infdis/jis632

7. Zhang F, Qi Y, Harrison TJ, Luo B, Zhou Y, Li X, et al. Hepatitis E genotype 4 virus from feces of monkeys infected experimentally can be cultured in PLC/PRF/5 cells and upregulate host interferon-inducible genes. *J Med Virol.* (2014) 86(10):1736‑44. doi: 10.1002/jmv.24014

8. Choi YH, Zhang X, Tran C, Skinner B. Expression profiles of host immune response-related genes against HEV genotype 3 and genotype 1 infections in rhesus macaques. *J Viral Hepat*. (2018) 25(8):986‑95. doi: 10.1111/jvh.12890

9. Todt D, Friesland M, Moeller N, Praditya D, Kinast V, Brüggemann Y, et al. Robust hepatitis E virus infection and transcriptional response in human hepatocytes. *Proc Natl Acad Sci U S A*. (2020) 117(3):1731‑41. doi: 10.1073/pnas.1912307117

10. Rusinova I, Forster S, Yu S, Kannan A, Masse M, Cumming H, et al. INTERFEROME v2.0: an updated database of annotated interferon-regulated genes. *Nucleic Acids Research.* (2012) 41(D1):D1040‑6. doi: 10.1093/nar/gks1215
